# Gamma Irradiation of Magnetoresistive Sensors for Planetary Exploration

**DOI:** 10.3390/s120404447

**Published:** 2012-04-02

**Authors:** Ruy Sanz, Ana B. Fernández, Jose A. Dominguez, Boris Martín, Marina D. Michelena

**Affiliations:** Space Programs & Space Sciences Department of the Spanish National Institute of Aerospace Technology (INTA), 28850, Torrejón de Ardoz, Madrid, Spain; E-Mails: cu4570_28@inta.es (R.S.); fernandezdab@inta.es (A.B.F.); cu4570_27@inta.es (J.A.D.); bmartin@slice-e.com (B.M.)

**Keywords:** magnetic devices, radiation effects in devices, Gamma ray effects, space applications, space radiation effects

## Abstract

A limited number of Anisotropic Magnetoresistive (AMR) commercial-off-the-shelf (COTS) magnetic sensors of the HMC series by Honeywell, with and without integrated front-end electronics, were irradiated with gamma rays up to a total irradiation dose of 200 krad (Si), following the ESCC Basic Specification No. 22900. Due to the magnetic cleanliness required for these tests a special set-up was designed and successfully employed. Several parameters of the sensors were monitored during testing and the results are reported in this paper. The authors conclude that AMR sensors without front-end electronics seem to be robust against radiation doses of up to 200 krad (Si) with a dose rate of 5 krad (Si)/hour and up to a resolution of tens of nT, but sensors with an integrated front-end seem to be more vulnerable to radiation.

## 1. Introduction

Magnetic sensors have attained considerable importance as part of the positioning and compassing systems in the field of automation, and their use today ranges anywhere from ground vehicles to aerospace platforms and devices such as cell phones and video consoles. Miniaturization and power consumption are key parameters for a successful integration in all these applications.

When miniaturization is an issue, miniaturized solid state sensors for magnetic sensing are often the common choice. Among the different types of solid state sensors, since the nineties magnetoresistive sensors have been proven to be the sensors with the highest technology readiness level (TRL) in the market. Due to their lower power consumption and weight, magnetometers based on AMR technology (as opposed to fluxgate, cesium or proton magnetometers) have been successfully employed on-board Unmanned Aerial Vehicles to perform geomagnetic surveys in extreme condition areas [1] with 7 nT resolutions. However, the requirements for space mission applications are more demanding [2]. Sensor radiation resistance is one of the main concerns when designing optimal scientific instruments to be embarked on space platforms.

Previous space missions [3] used AMR COTS magnetic sensors for different applications: experimental, Attitude and Orbital Control Systems (AOCS), *etc.* Consequently, upscreening tests for these sensors, including radiation tests, were performed [4]. This work and others [5] seem to agree that AMR and Permalloy (Py) based sensors are not damaged by gamma radiation up to a total irradiation dose (TID) of up to 100 krad. Other authors have speculated with potential damage caused by protons associated with the defects created in the Permalloy during bombardments [6,7], but more tests should be carried out in order to arrive at conclusive data.

In this work, a systematic gamma irradiation test of the AMR sensors already used in INTA nanosatellites (one-axis magnetoresistive Wheatstone bridge) was performed, as well as on some AMR COTS sensors with more axes and with part of the front-end integrated in an Applied Specific Integrated Circuit (ASIC). Gamma irradiation tests are usually performed on materials and devices which have the same response in the external magnetic field. In the present case, special care was taken to ensure the magnetic cleanliness of the environment during the characterization. All the tests were carried out assuring low disturbances of varying magnetic fields, keeping the variations under the error threshold by means of magnetic shielding, and registering magnetic field variations with a pT resolution. The objective was twofold: study the damage of these sensors with TID, and in case of failure try to discern the part of the sensor responsible for such a failure.

Results on the degradation of four AMR sensors when irradiated with gamma rays up to a TID of 200 krad are presented in this paper and described in the next section [8]. Parameters such as linear response and saturation field, offset and set/reset strip deviations and power consumption were monitored for the four different types of sensors during the irradiation with powered and non-powered units. The radiation envelope requirement for the MetNet precursor mission is 15 krad. However, the objective was to validate these sensors to be able to use them as space weather sentinels in the L1 Lagrange point, together with miniaturized particle detectors and other radiation sensors. Hence, the tested sensors were exposed to an extended gamma radiation TID of 200 krad, and testing included a sufficient number of steps so as to be representative of the radiation dose of other current missions: OPTOS and MetNet.

## 2. Experimental Section

### 2.1. Device Basis

The sensors chosen for the test were the HMC series by Honeywell: HMC 1021 S (one axis and no front-end), HMC 1043 (1043 lot: 2010014408 batch: c5x405620, three axes and no front-end), HMC 6042 (two axes and amplifiers) and HMC 6052 (two axes with amplifiers and part of the set/reset needed circuitry). The HMC1021 sensor had been already used in the AOCS of INTA nanosatellites NANOSAT-01, NANOSAT-1B [3] and picosatellites like DTU-Sat [9]. HMC 1043 was chosen for the AOCS of the INTA OPTOS picosatellite and as the magnetic sensor payload for the first lander of the MetNet Precursor mission to Mars, which is supposed to be the first penetrator and ground-based meteorological station on Martian surface [10] capable of registering magnetic field variations generated by crustal minerals due to temperature variations [11]. The payloads in this mission have a very limited power consumption and mass (150 g for the three Spanish payloads: an irradiance sensor, a dust deposition sensor and a three axes magnetometer with a gradiometer), which requires the use of AMR COTS technology for the magnetometer.

**Figure 1.**
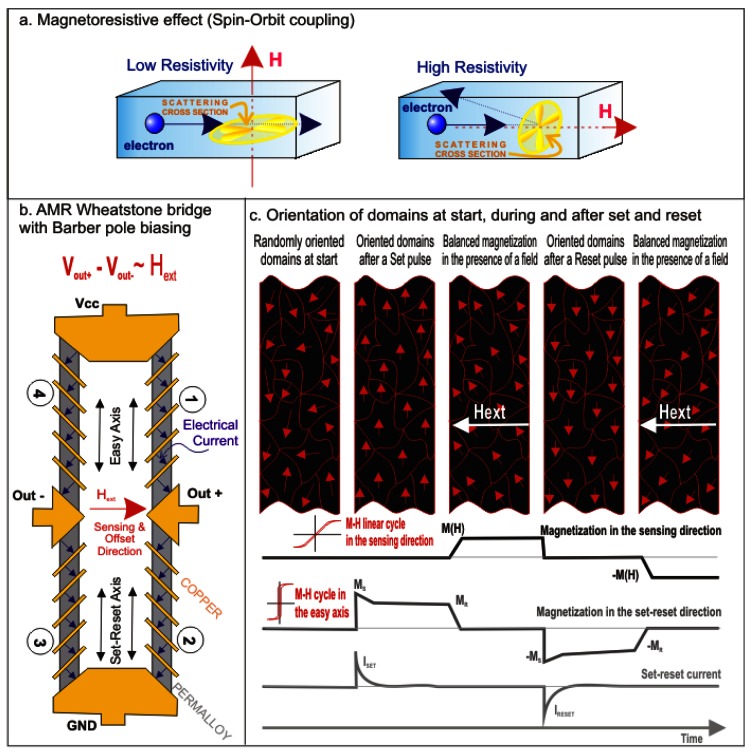
(**a**) Magnetoresistive effect; (**b**) AMR Wheatstone bridge with Barber pole biasing; and (**c**) Sequential orientation of the spins in the domains at the start, during and after a set and during and after a reset.

The technology of the AMR sensors tested was CMOS. They consist of Wheatstone bridges to measure magnetic fields. Magnetoresistance is the variation of the electrical resistance of a material when it is immersed in a magnetic field, and is a consequence of spin orbit coupling. The electronic clouds of atoms tend to be distributed in a plane perpendicular to the field, so the scattering of transport electrons (electrical current) differs, depending on the angle there is between the magnetization in the material (which follows the external field) and the direction of the electrical current (Figure 1(a)). In fact, the effect is proportional to the squared cosine of the angle formed by the two directions (Figure 1(a)). Graphically, it can be thought of as a measure of the projection of the magnetization of the film over the direction of the current, and in principle it is a even effect, which makes it impossible to distinguish between positive and negative fields. The magnetoresistive elements of the bridge are patterned permalloy (py) thin-films acting as a resistive strip (Figure 1(b)). Py, a Fe and Ni alloy is a well known magnetic material with very low magnetocrystalline anisotropy; hence, shape anisotropy contribution has a main role in the total anisotropy [12]. The Py strips easy-axis was oriented along one specific direction during the manufacturing of the device. The sensing direction of the sensors is perpendicular to that of the easy axis. In the sensors tested, a barber pole biasing [8] was used to provide an odd response of the sensor versus the applied magnetic field, making the electrical current circulate at a 45° angle with respect to the easy axis and the sensing direction by means of copper straps at −45° along the thin film. These sensors have the best sensitivity when magnetization is rotated from the easy axis to a stable direction, balancing the anisotropy and the external magnetic field. By doing so, and thus increase the repeatability of the measurements and robustness against moderate to high (50 μT) magnetic fields exposure, the sensors were equipped with the so-called set/reset strap: a coil generating a restoring magnetic field in the easy axis prior to the measurement (Figure 1(c)). In addition, sensors are equipped with offset straps, with the twofold objective of performing calibrations or using them in a closed loop to make measurements in the zero field area [8]. In these tests, offset coils as well as external Helmholtz coils were used to apply linear variations of the magnetic field in order to obtain additional measurements (internal coils configuration) up to absolute magnetic fields of 200 μT (HMC 1021 S) and 100 μT (rest of sensor family).

From the device point of view, the test focused on the sensitivity and offset of the sensors, and the behavior of the two internal coils: set/reset and offset straps.

The offset is the response of the sensor in the absence of a magnetic field. It is a measure of the imbalance of the resistors of the bridge. Although they are laser trimmed, normally there is a slight difference among the values, which makes the Wheatstone bridge output imbalanced even when no external field is applied. Offset changes during the irradiation procedure can be due to changes in the values of the four resistors. The higher the imbalance between values, the greater the influence this effect would have. The offset is dependent on temperature. Thus, this parameter needs to be controlled. Since it is very difficult to get a zero field environment (even more in the irradiation facility), during the test, the environmental magnetic field in the position of the sensors is controlled.

Sensitivity is the derivative of the output voltage respect to the external magnetic field. It can change if there are asymmetries in the set and reset response, if the offset changes and it is also temperature dependent. The sensitivity was measured by applying a ramped field with external coils inside the magnetic field chamber. With these ramps the linear range was also measured.

Each sensor was always measured in set-reset mode. The set-reset current peak was randomly measured in some of the measurements to ensure that the set/reset strap resistance did not change. This is important because the set-reset strap is supplied with voltage.

The offset strap efficiency *i.e.*, the magnetic field generated as a function of the electrical current, was measured in comparison with the ramp generated with external coils. Thus, the offset coils were supplied with a current source.

### 2.2. HMC1021 and HMC1043 Specifications

Sensors HMC 1021 S and HMC 1043 are based on the same architecture consisting of an AMR Py film Wheatstone bridge with barber pole biasing. HMC 1021 S is a one-axis sensor and HMC 1043 is a 3-axes sensor. Sensors had the integrated offset coil and the set/reset coil described above. In the case of the HMC 1043, the three Wheatstone bridges shared the connection to the power source and the offset and set/reset straps of the x and y axes were connected in series inside the device, the separated pins not being accessible. Specifications are summarized in Table 1 [8].

### 2.3. HMC6042 and HMC6052 Specifications

HMC 6052 and HMC 6042 chips are two axis sensors with incorporated ASICs for signal conditioning. In the HMC 6052 the ASIC consists of instrumentation amplifiers for the Wheatstone bridges signals and in the HMC 6042 it consists of the instrumentation amplifiers and part of the set/reset circuitry. Some of the specifications extracted from [8] are summarized in Table 1.

### 2.4. Irradiation Test Plan

The irradiation was carried out in April 2011 with a Co-60 source at the Radiophysics Laboratory (Universidad de Santiago de Compostela, Spain). The irradiation test plan was designed to fulfill the ESCC Basic Specification No. 22900 [13] requirements, but was carried out only on a small sample of sensors. Final annealing, as indicated in the specification process (128 °C, 24 hours), was not carried out because on the one hand, the irradiation rate was very high and was thus a worst case for the CMOS devices, and on the other hand very low damage was seen in the devices. However, a couple of measurements were performed 24 and 48 hours after the last irradiation step at a higher temperature, due to the different geographic locations (Santiago de Compostela: 42°52′N and 8°32′W; and Madrid: 40°23′N and 3°43′W). The components tested were: two HMC 1021 S units, four MHC 1043 units, four HMC 6042 units and four HMC 6052 units. Half of the devices of each type were powered during irradiation and the other half were neither powered nor connected to ground. The irradiation test plan is summarized in Table 2. The time between irradiation and measurements was always limited to 2.5 hours. TID and dose rate were calculated to induce high damage to the CMOS technology [14,15].

### 2.5. Set-Up

For the sake of simplicity, all the sensors tested were soldered to a 2.5 cm^2^ Printed Circuit Board (PCB) with soldered pins for easy connection of the sensors to the test board. These test boards were unique for each type of sensor. This connection method was selected since it reduces sensor alignment errors in repeated measurements. The number of sensors irradiated at the same time and the absence of magnetic cleanliness in the gamma-ray source facility made it impossible to test sensors *in-situ*, so the pinned PCB made it easier to make measurements in “remote” configurations [13]. During the irradiation, the sensors were connected to a PCB with power supply and ground lines for the powered units.

**Figure 2.**
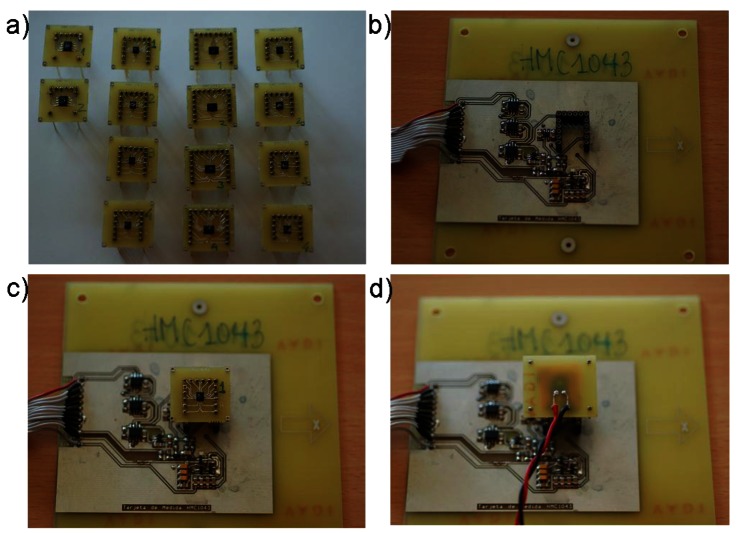
(**a**) From left to right in columns: sensors HMC 1021 S, HMC 1043, HMC 6042, HMC 6052 soldered to PCBs; (**b**) Test board of the HMC 1043 sensor; (**c**) Sensor HMC 1043, number 1, plugged to the test board; and (**d**) HMC 1043 and PT-1000 plugged to the test board.

After each irradiation step, the sensors were plugged to the test board (see Figure 2), and altogether introduced in a three-layer magnetic shielding chamber located in an area of the facility with a minimum magnetic field and field gradient for the different parameter measurements. Every sensor was measured at the beginning of the test in the same location. To compensate for the variations of the Earth magnetic field and temperature, a couple of reference magnetic and temperature sensors were used. The magnetic field inside the chamber was monitored before each test by a calibrated 3-axis fluxgate magnetometer of tens of pT resolution (Mag-03MSL500 by Bartington, UK) and the measurements of the different magnetic sensors were correlated with those of the fluxgate, which registered components of the magnetic field with standard deviations of: Δx = 38 nT, Δy = 31 nT, Δz = 43 nT. However, occasional variations in the intensity of the environmental field of moderate intensity (up to 100 nT) were observed outside of the magnetic field chamber during the measurements after 7, 15, 50, 100 and 200 krad steps. The corresponding maximum expected variation of the field inside the chamber is attenuated by a factor of 7. For temperature compensation, a piggy back Platinum resistor PT-1000 was placed on top of the AMR sensor in every measurement, and the change of the resistance of the PT-1000 was monitored and acquired as one more parameter by a millimeter Agilent 34401A. The AMR sensors theoretical variation of sensitivity with temperature was used [8].

**Figure 3.**
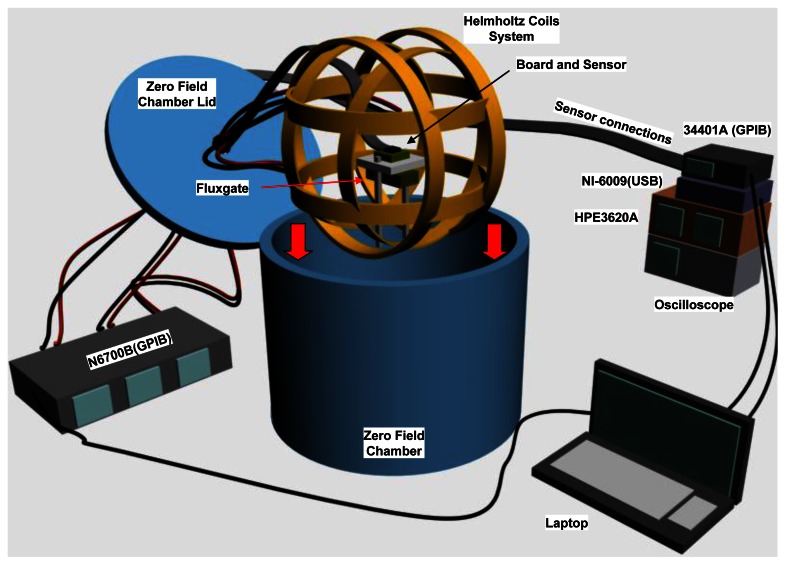
Sketch of the measurement set-up. Types of computer connections are indicated in brackets.

The remaining elements of the set up for remote measurements after each irradiation step (Figure 3) are described below:

#### 2.5.1. Test Boards

For each sensor model a specific test board was developed. The PCBs supplied the voltage bridge by means of a voltage precision reference of 3.3 V. For HMC 1021 S and HMC 1043, the test PCB consisted of the front-end electronics with amplifiers and set/reset circuitry. HMC 6052 PCB uses a voltage reference as power supply and the HMC 6042 board feeds internal conditioning electronics with a voltage regulator. The outputs of the sensors were amplified by means of an AD627 instrumentation amplifier. Power consumption of the whole set (AMR sensor and PCB) was measured by means of a HPE3620A DC power supply unit. This is justified because magnetoresistors are the most dissipative elements in the PCB.

#### 2.5.2. Helmholtz Coils and Magnetic Shielding Chamber

In order to obtain a homogeneous and controlled magnetic environment, the system of coils was centered in a shielding chamber of CO-NETIC AA alloy (Magnetic Shield Corp., USA) which attenuates the external magnetic field (factor 10^4^). As previously mentioned, the magnetic field inside the chamber was measured before each test by the fluxgate magnetometer. Inside the chamber the magnetic field is generated by means of 3 pairs of Helmholtz coils with a tradeoff diameter size between the internal layer of the shielding chamber and the size of the test boards. The size of the coils is much smaller than that of the internal shielding layer, so as to not be affected by the shielding alloy contribution but higher than the test board in order to have a uniform magnetic field in all points of the PCB. The expected misalignment of the generated magnetic field and the sensor measuring axis was less than 1°, which corresponds to a variation of the field of 0.15‰. A N6700B Agilent supplied the current necessary for the magnetic field in every direction. In this way, a homogeneous magnetic field of up to 600 μT was generated in the geometrical center of the system, where the test board was held by a non-magnetic plastic (PVC) holder.

#### 2.5.3. Data Acquisition and Additional Test Equipment

Data acquisition processes and set/reset digital signals were carried out. The acquisition was performed with a National Instruments NI-6009 USB Digital/Analogic data acquisition unit. The software was able to measure the response of the sensor when a magnetic field was ramped in the sensing directions. In order to assure the proper measurement state of the sensors, the set/reset pulses were monitored (intensity and full width at half maximum—FWHM) and recorded by means of an oscilloscope (Agilent DSO 6014A).

## 3. Results and Discussion

As already mentioned in Section 2.1, four different parameters were measured at every step of the test:

- the linear response of the sensors by means of its offset and sensitivity values and the bridge voltage
- the correct reset of the sensor by the peak measurement the of current in the offset straps
- the efficiency of the offset straps by comparison with the field generated by external coils
- the power consumption, because variations in power consumption can be related to a malfunctioning of the sensor and the conditioning electronics.

During testing there were no noticeable changes in power consumption. In general, the sensors exhibited a very low degradation with the TID, lower than the measurement error: 2% (limited by the environment and the instrumentation used). The observed sensitivity variations, measured as the percentage of deviation with respect to initial values, and the absolute variation of the offset for each type of sensor are described in the following paragraphs. The offset values were obtained by linear fitting of sensor responses. The reason to do this is that there is a certain bias field due to residual currents in the circuit, and thus the fit (with a variation of less than 1 nT with respect to the measured value) is considered to be a better measurement. The mean maximum uncertainty value for sensor offset was taken as the resolution declared by the manufacturer, *i.e.*, 12 nT. This criterion is justified because the moderate variations in the intensity of the magnetic field measured by the reference fluxgate (reported in Section 2.4) were taken into account. Measurements performed 24 and 48 hours after the last irradiation step are denoted by 200* and 200** in the TID x-axis. Error bars include the propagation of errors through the electronic chain, variations in temperature and the misalignment between Helmholtz coils and sensor axes. Notice that some of the missing measurements of HMC 1021 S and HMC 1043 at a TID of 200 krad are due to a malfunctioning of a common electronic component in the test boards and are not attributable to the sensors.

### 3.1. HMC 1021S

**Figure 4.**
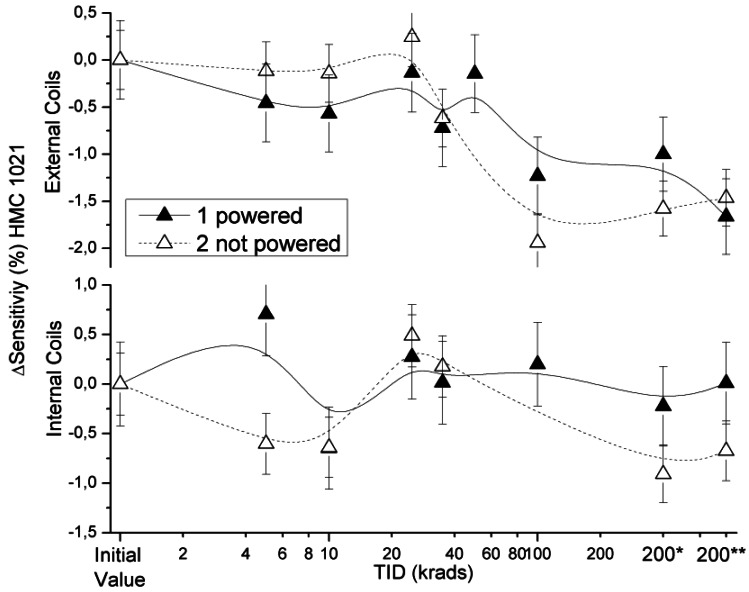
Sensitivity deviation measured for external and internal coil configuration as a function of TID for powered and non-powered HMC 1021 S.

The effects of radiation on the HMC1021S sensitivity measured by the external and internal coils are presented in Figure 4. Percentage sensitivity variation refers to the initial sensitivity. The variations measured by means of the external Helmholtz coils were below 2%, as it can be seen in the graph. A slight drift towards lower sensitivities can be observed. This is attributed to a slight movement of the relative position between the sensor and the coils during the 200* and 200** measurements. The sensitivity of the offset strap of the former value measured also had a variation lower than the 2%, which is attributable to experimental errors. However, the average of the measurements during testing was constant, which supports our previous assumption.

**Figure 5.**
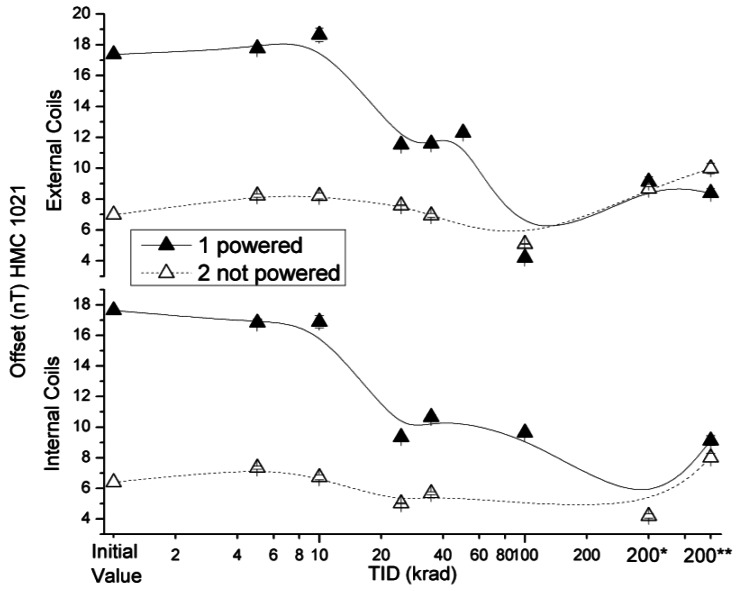
Measured offsets in external and internal coils configuration as a function of TID for powered and non-powered HMC 1021 S.

The radiation effects on the HMC 1021 S offset values under the external and internal coils configuration are presented in Figure 5. The variations of the offset in sensors in both configurations measured were below the uncertainty value declared by the manufacturer, except for the powered sensor after a TID of 100 krad. However, the 2 nT extra deviation is not considered to be a conclusive damage and is probably due to a measurement error. This conclusion is reinforced by the HMC 1043 data described in Section 3.2.

### 3.2. HMC 1043

Due to the aforementioned problem, with a component included in the test board (MOSFET employed for the external application of set/reset pulse) non-powered sensor number 3 suffered an overload and was damaged during testing. The substitution of the said component did not compromise the measurements of the other sensors since they were not affected by the overload.

**Figure 6.**
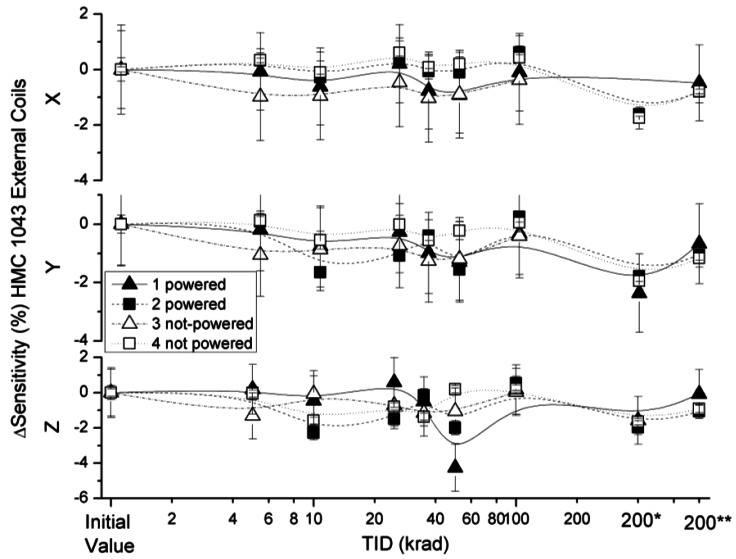
Sensitivity deviation measured for external coil configuration as a function of TID for powered and non-powered HMC 1043.

**Figure 7.**
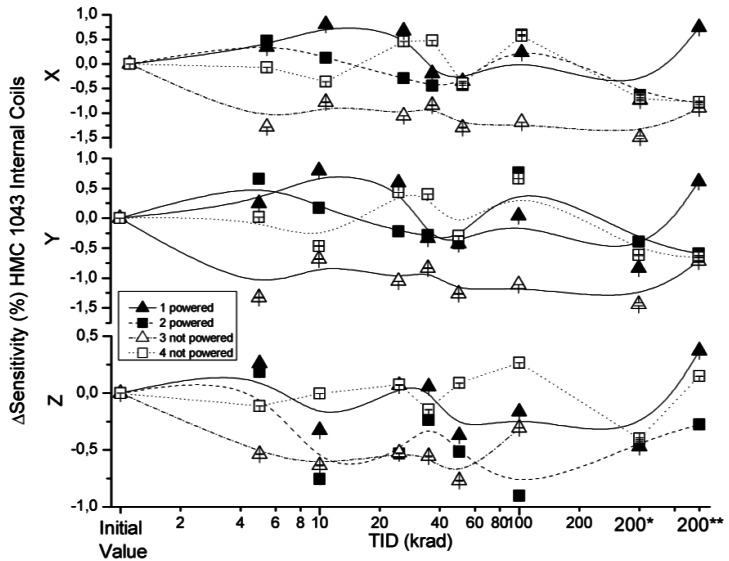
Sensitivity deviation measured for internal coils configuration as a function of TID for powered and non-powered HMC 1043.

The effect of radiation on sensitivity in the x, y and z axes of the HMC 1043 measured with external and internal coil configurations are presented in Figures 6 and 7. Sensitivity variations under external coils configuration were below 3%, with the exception of the non-powered sensor (number 3) and a powered sensor (number 1) for a TID of 45 krad. Sensitivity variations obtained in the internal configuration had values below 1.5%. Sensor sensitivity in both configurations was measured with a time interval of less than 5 minutes, which leads to the conclusion that the higher deviation measured for certain TID of the HMC 1043 with the external coils configuration was due to a misalignment of the sensor during testing.

**Figure 8.**
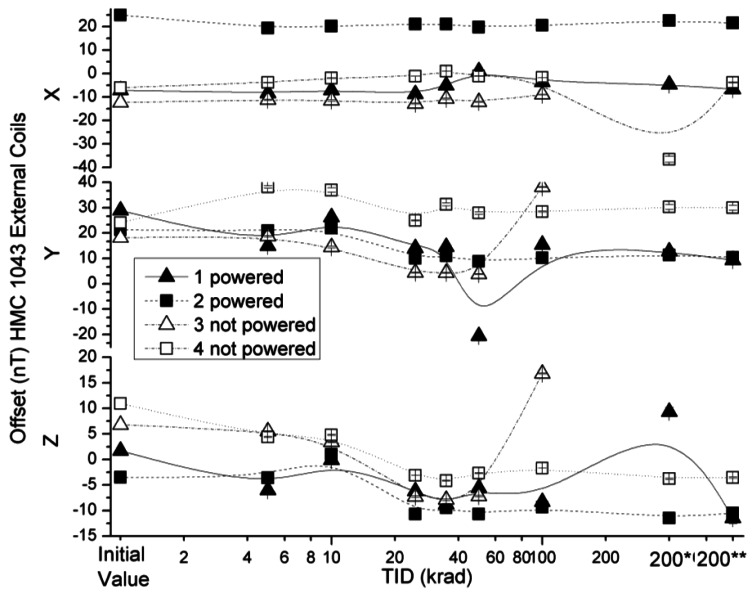
Measured offsets in external coils configuration as a function of TID for powered and non-powered HMC 1043.

The effects of radiation on absolute offset values of x, y and z axes of the HMC 1043 under external coil configuration are presented in Figure 8. The variations of the offset in powered sensors in both measured configurations were below the assumed uncertainty value, except for the previously mentioned exceptions of sensors 1 and 3. However, non-powered sensors have higher variations than these values. The obtained offset values (data not shown) in internal coil configuration were under the uncertainty value declared by the manufacturer.

### 3.3. HMC 6042

**Figure 9.**
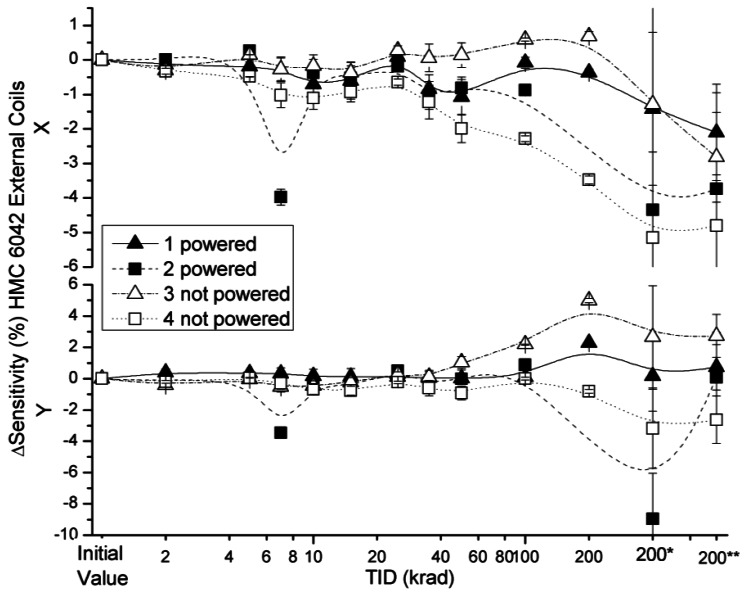
Sensitivity deviation measured for external coil configuration, as a function of TID for powered and non-powered HMC 6042.

**Figure 10.**
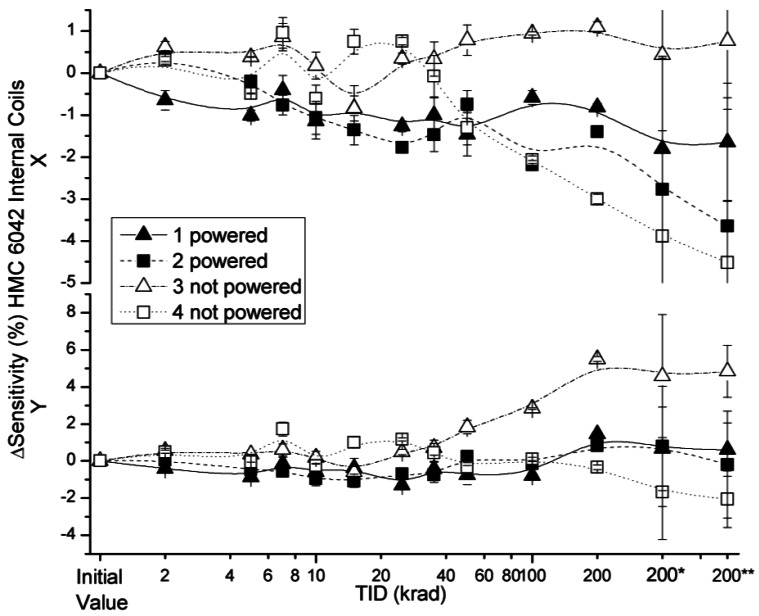
Sensitivity deviation measured for internal coil configuration as a function of TID for powered and non-powered HMC 6042.

The effects of radiation on sensitivity for the x and y axes of HMC 6042 in external and internal coils configurations are presented in Figures 9 and 10, respectively.

Sensitivity deviations measured with both configurations were below 3%, with the exception of two powered and non-powered sensors (numbers 2 and 4) after a TID of 200 krad during 200* and 200** measurements. A certain decrease in sensitivity was expected due to the higher temperature of the measurements of the annealing, which justifies the higher length of the error bars in these measurements. Although in the two aforementioned sensors the sensitivity variation is not compatible with the previous measurements despite the higher uncertainty in the measurement, they are attributed to variations in the set up because no damage was observed during the irradiation. The same conclusion is extracted with respect to the peak observed in the powered device number 2 in the 7 krad step. Actually, this peak can correspond to the aforementioned quick change in the environmental field at several steps of the irradiation. One interesting result is the drift of the sensitivity variation with internal coils that can be observed in Figure 10 in the x axis for devices number 1, 2 and 4. No explanation was found for this observation.

**Figure 11.**
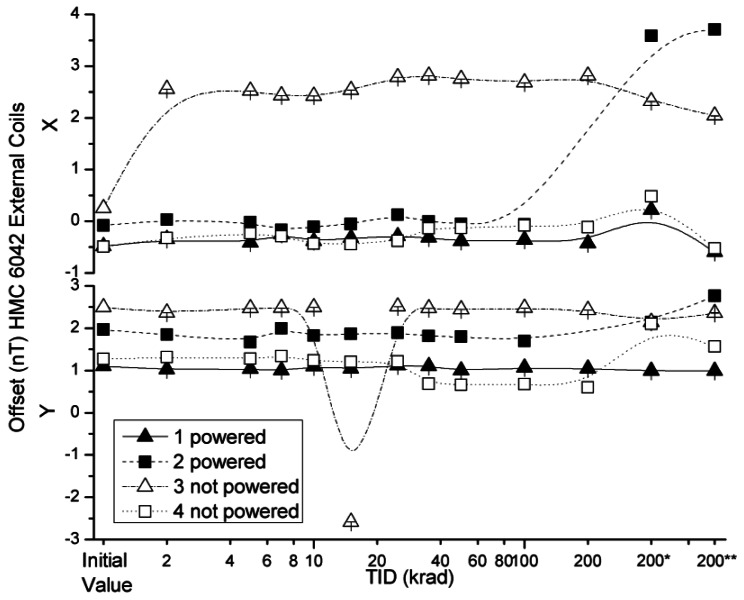
Measured offsets in external coils configuration as a function of TID for powered and non-powered HMC 6042.

**Figure 12.**
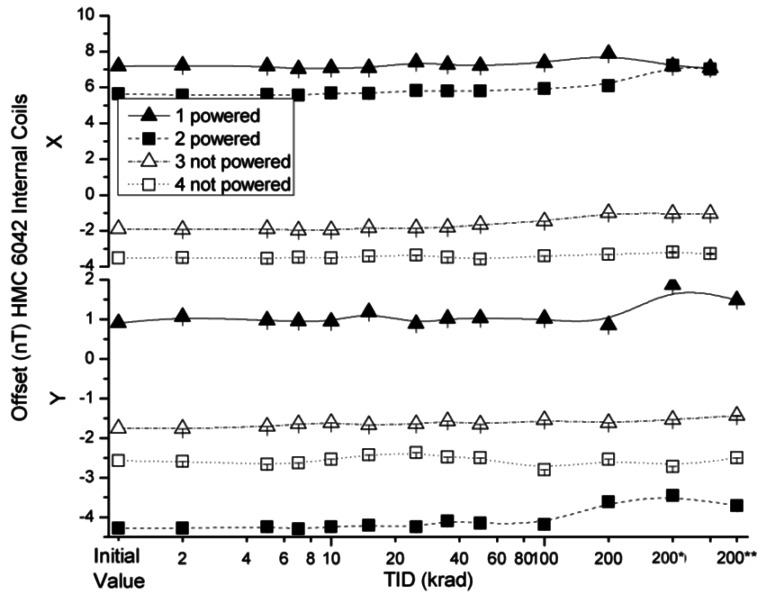
Measured offsets in internal coils configuration as a function of TID for powered and non-powered HMC 6042.

The effects of the radiation on absolute offset values of the HMC 6042 x and y axes with external coil and internal coil configurations are presented in Figures 11 and 12, respectively. The variations of the offset in both measured configurations were below the uncertainty value declared by the manufacturer, with minor deviations for one measurement of sensor number 3 and two measurements of sensor number 4. These results may indicate the appearance of some build-up of interface states and would need further testing with a collimator in order to discern if they are produced by the front-end integrated ASIC.

### 3.4. HMC 6052

**Figure 13.**
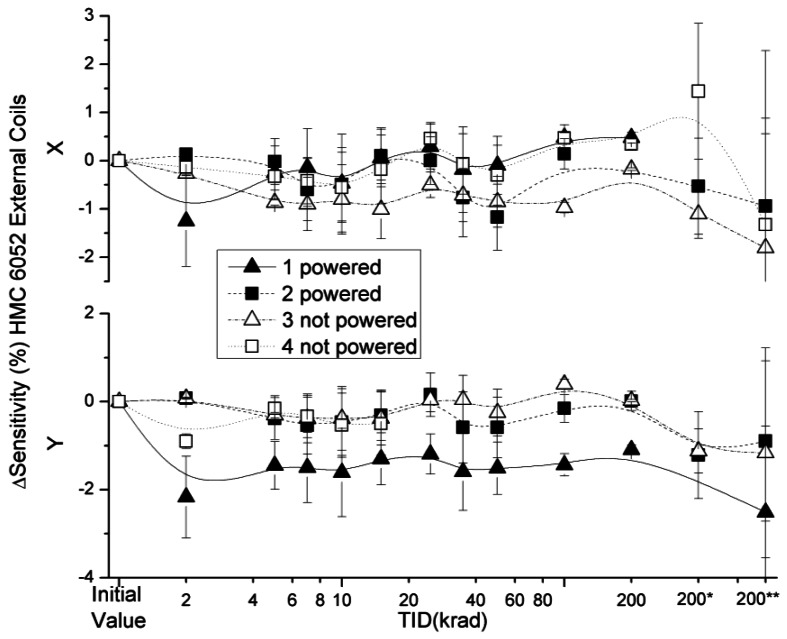
Sensitivity deviation measured for external coils configuration as a function of TID for powered and non-powered HMC 6052.

**Figure 14.**
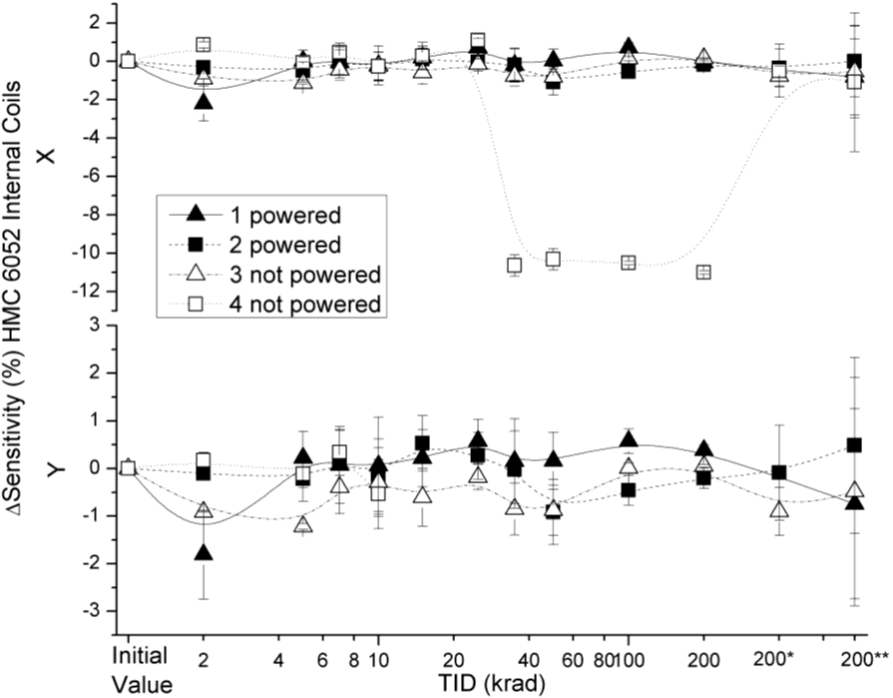
Sensitivity deviation measured for internal coils configuration as a function of TID for powered and non-powered HMC 6052.

The effect of the radiation on the sensitivity in the HMC 6052 x and y axes with external and internal coil configurations are presented in Figures 13 and 14. Variations in sensitivity were below 3% with the exception of a non-powered sensor (number 4). The signals from the y axis and x axis of this sensor started to oscillate at TID of 2 krad and 25 krad, respectively. After 15 krad it was not possible to register a signal from the y axis. However, during 200* and 200** measurements the x axes of the sensor resumed their functioning under standard parameters; the high dispersion of the values measured with this sensor led us to believe that it was either badly soldered or seriously damaged during the irradiation process. To discern if the damage could be in the ASIC (very plausible), a future irradiation could be performed on the single amplifier of the ASIC to see if it reaches an oscillatory behaviour.

**Figure 15.**
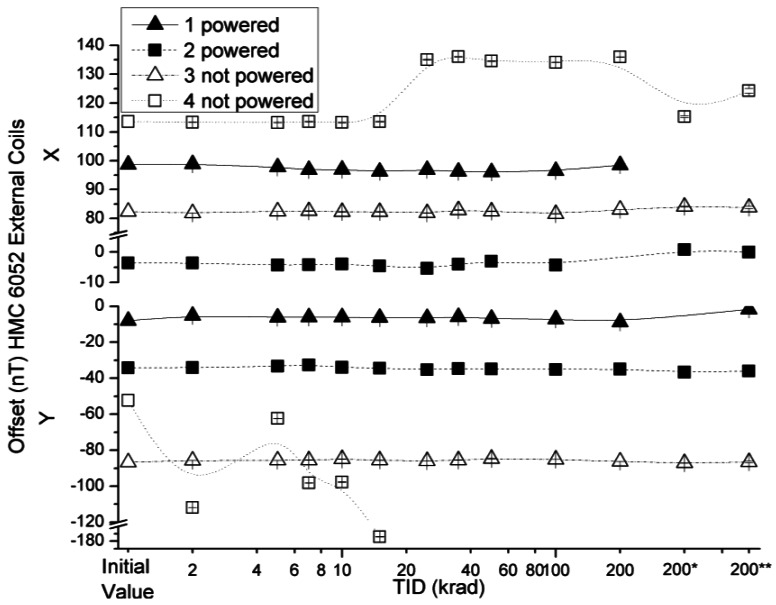
Measured offsets in external coils configuration as a function of TID for powered and non-powered HMC 6052.

**Figure 16.**
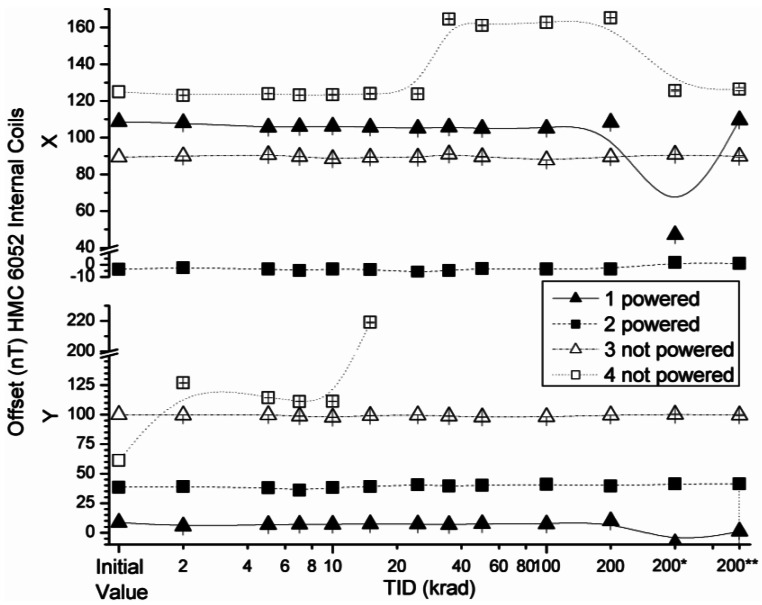
Measured offsets in internal coils configuration as a function of TID for powered and non-powered HMC 6052.

The effect of the radiation on the offset absolute values of the HMC 6052 x and y axes with external coils and internal coils configurations are presented in Figures 15 and 16, respectively.

The variations of the offset in both measured configurations were below the uncertainty value declared by the manufacturer, with the exception of the aforementioned non-powered sensor number 4. The fact that the polarity of the y component in the measurement with internal coils and external coils is inverse is noteworthy. This is due to the following reason: the PCB for the measurements is the same for all the sensors, as described before, and its axes had been chosen as a right-handed coordinated system. During testing, all x axes of the sensors aligned with the x axis of the PCB, but this was not the case of the HMC6052 sensor, whose y axis pointed to the −y axis of the PCB.

The observed effects of gamma irradiation on the sensors tested can be due to damages in the Py strips or in the front-end electronics. Regarding Py strips, gamma irradiation will only affect the Py magnetic response if enough electrons are displaced at higher energy levels and therefore change the magnetic ordering of the material [5], but the displaced electrons would immediately be restored when a magnetic a field is applied. However, changes in sensor sensitivities can be observed with the increase of TID. These changes in sensitivity are more noticeable for non-powered devices than for the powered ones. Non-powered sensors were not ground connected during gamma irradiation. This may have induced an accumulation of charges, and therefore it could induce dielectric breakups/electrostatic discharges in the front-end electronics.

## 4. Conclusions

With a view to their future use for planetary missions, several types of AMR COTS sensors were irradiated with gamma rays up to a TID of 200 krad: HMC 1021 S, HMC 1043, HMC 6042 and HMC 6052.

- HMC 1021 S sensors had low degradation both in sensitivity (<2%) and offset values (<12 nT).
- The HMC 1043 triaxial magnetic sensor tested had a low degradation up to a TID of 100 krad gamma irradiation. Offset values had low deviations up to 200 krad (<12 nT), and sensitivity of HMC 1043 had low degradation (<5%) under gamma irradiation up to 100 krad. However, after a TID of 200 krad a non-powered sensor exhibited marked variations. The test performed points out the suitability of sensor HMC 1043 to fulfill gamma irradiation requirements for a future Met-Net precursor mission to Mars.
- The HMC 6042 biaxial magnetic sensors tested had a low degradation response up to TID 100 krad, both in sensitivity variation (<3%) and offset absolute value (<2 nT). However, two sensors had increased deviations for TID of 200 krad and subsequent measurements.
- The HMC 6052 biaxial magnetic sensors tested had a low degradation response (<3%) both in sensitivity variation and offset absolute value (<2 nT) up to a TID of 200 krads and subsequent annealing. However, the failure of a non-powered sensor after a TID of 2 krad makes future testing necessary in order to derive more significant results.

It is concluded that AMR technology seems to be robust against TID up to 200 krad with an error down to 10 nT, and the sensors seem to behave better if they are powered. However, sensors with integrated ASIC's do not seem to have such high robustness. In our particular case, this has encouraged us to develop an ASIC for the electronic conditioning of AMR sensors. The implementation of the ASIC in AMR sensors will be of utmost importance for their miniaturization. Furthermore, their performance could be improved since the elements of the ASIC can be non-magnetic in order to guarantee magnetic cleanliness in the proximity of the transducer.

The fact that offset and set/reset traps were not affected by radiation is a very important conclusion because if sensors were damaged at higher doses or with different rates, it would always possible to perform an in-flight calibration by means of the offset straps.

Further work should focus on the following aspects:

1. To measure the noise of the sensors. This study has not been possible in the present case due to the limitation of the measurement error and the difficulty to control the magnetic cleanliness in the facility.
2. To elucidate if non-powered sensor reliance can be improved by ground connection during gamma irradiation to improve the physical effects on the straps.
3. To discern if changes in the behavior of the sensors with integrated ASIC's, namely HMC 6042 and HMC 6052, can be definitively attributed to the ASIC by means of a collimator or by a separate irradiation of the ASIC amplifiers.
4. To further increase the knowledge of AMR sensors under radiation for space applications, a future irradiation campaign with protons will be performed to see the effects of displacement damage in these components. In the magnetic sensor we foresee a higher influence of protons in the performance of the sensors than that observed with the total ionizing dose. As a result of the proton irradiation, we expect the appearance of defects in the Py that may hinder movement of the magnetic walls, increasing the hysteresis of the sensors and diminishing repeatability.

## Figures and Tables

**Table 1.** Summary of the properties of the different AMR sensors [8].

**Properties**	**HMC 1021 S**	**HMC 1043**	**HMC 6042**	**HMC 6052**
**Bridge Supply (V)**	5–25	1.8–20	2.4–2.6	2.5–2.6
**Field Range (10^5^nT)**	−6 to 6	−6 to 6	−6 to 6	−2 to 2
**Maximum Linear error (% FS)**	1.6	1.4	0.80	0.4 (±0.5 G)
**Resolution (nT)**	8.5 (10 Hz, 5 V)	12 (10 Hz, 5 V)	12 (1 kHz, 3 V)	8 (1 kHz, 3 V)

**Table 2.** Summary of the irradiation test plan.

**Step**	**Dose rate (krad(Si)/hour)**	**Dose/step (krad(Si))**	**TID (krad(Si))**	**Measured Sensors**
1	5	2	2	6042/6052
2	5	3	5	1021/1043/6042/6052
3	5	2	7	6042/6052
4	5	3	10	1021/1043/6042/6052
5	5	5	15	6042/6052
6	5	10	25	1021/1043/6042/6052
7	5	10	35	1021/1043/6042/6052
8	5	15	50	1021/1043/6042/6052
9	5	50	100	1021/1043/6042/6052
10	5	100	200	1021/1043/6042/6052
